# Toxicity of polymyxins: a systematic review of the evidence from old and recent studies

**DOI:** 10.1186/cc3995

**Published:** 2006-02-13

**Authors:** Matthew E Falagas, Sofia K Kasiakou

**Affiliations:** 1 Alfa Institute of Biomedical Sciences (AIBS), Athens, Greece; 2 Department of Medicine, 'Henry Dunant' Hospital, Athens, Greece; 3 Department of Medicine, Tufts University School of Medicine, Boston, Massachusetts, USA

## Abstract

**Background:**

The increasing problem of multidrug-resistant Gram-negative bacteria causing severe infections and the shortage of new antibiotics to combat them has led to the re-evaluation of polymyxins. These antibiotics were discovered from different species of *Bacillus polymyxa *in 1947; only two of them, polymyxin B and E (colistin), have been used in clinical practice. Their effectiveness in the treatment of infections due to susceptible Gram-negative bacteria, including *Pseudomonas aeruginosa and Acinetobacter baumannii*, has not been generally questioned. However, their use was abandoned, except in patients with cystic fibrosis, because of concerns related to toxicity.

**Methods:**

We reviewed old and recent evidence regarding polymyxin-induced toxicity by searching Pubmed (from 1950 until May 2005).

**Results:**

It was reported in the old literature that the use of polymyxins was associated with considerable toxicity, mainly nephrotoxicity and neurotoxicity, including neuromuscular blockade. However, recent studies showed that the incidence of nephrotoxicity is less common and severe compared to the old studies. In addition, neurotoxic effects of polymyxins are usually mild and resolve after prompt discontinuation of the antibiotics. Furthermore, cases of neuromuscular blockade and apnea have not been reported in the recent literature.

**Conclusion:**

New evidence shows that polymyxins have less toxicity than previously reported. The avoidance of concurrent administration of nephrotoxic and/or neurotoxic drugs, careful dosing, as well as more meticulous management of fluid and electrolyte abnormalities and use of critical care services may be some of the reasons for the discrepancy between data reported in the old and recent literature.

## Introduction

Polymyxins were discovered in 1947 from different species of *Bacillus polymyxa *[1,2]. Although the effectiveness of polymyxins against most Gram-negative bacteria, including *Pseudomonas aeruginosa *and *Acinetobacter baumannii*, has not been questioned, early administration of polymyxins was associated with reports of adverse renal and neurological effects in a considerably large number of patients [3,4]. Thus, compounds of this class of antibiotics were gradually withdrawn from clinical practice as newer antibiotics with the same or broader antibacterial spectra and reportedly lower toxicity were introduced, except for patients with cystic fibrosis who suffer from recurrent pulmonary infections due to multidrug-resistant bacteria [5-7]. However, the emergence of Gram-negative bacteria that are resistant to almost all classes of available antibiotics except polymyxins, especially *Pseudomonas aeruginosa *and *Acinetobacter baumannii *strains, and the shortage of new antibiotics with activity against them has led to the re-use of polymyxins [8-12]. The objective of this critical review of the old and recent literature is to elucidate the incidence, mechanisms, prevention, and treatment of adverse events of polymyxins, focusing on patients without cystic fibrosis.

This class of antibiotics consists of five chemically different compounds, polymyxin A, B, C, D, and E (colistin). Only polymyxins B and E have been used in clinical practice. Colistin consists of a cyclic heptapeptide and a tripeptide side-chain acylated at the amino terminus by a fatty acid. The amino acid components in the molecule of colistin are D-leucine, L-threonine, and L-α-γ-diaminobutyric acid. Polymyxin B has the same structure as colistin but contains D-phenylalanine instead of D-leucine [13].

Commercially, colistin appears as colistin sulfate, which is used orally for bowel decontamination and topically as a powder for skin infections, and as colistimethate sodium, which is used parenterally and by inhalation. Colistimethate sodium has been found to be less toxic and to have fewer undesirable side effects than colistin, but is also less potent. Polymyxin B is available for clinical use as polymyxin B sulfate and is used parenterally, topically (ophthalmic and otic instillation), intrathecally, by inhalation, and as an irrigation solution [14,15].

Several attempts to generate less toxic derivatives were made [16]. Most of these derivatives lacked the fatty acid and/or the diaminobutyric acid components of their original molecules. Experimental studies demonstrated that these compounds were much less toxic compared to the parent ones, but at the same time they had considerably reduced antibacterial effect [17,18].

## Methods

Data for this review were obtained through literature searches of publications included in PubMed from 1950 until May 2005, references cited in relevant articles, and the world-wide web. The main search terms used in searches of literature databases were 'colistin', 'polymyxin E', 'polymyxin B', 'adverse effects', 'nephrotoxicity', 'colomycin', 'colimycin', 'neurotoxicity' and 'toxicity'. Only English language papers were reviewed.

## Results and discussion

In Tables 1 and 2 we summarize the available publications reporting data regarding the incidence of toxicity, including nephrotoxicity, neurotoxicity, and other adverse effects of polymyxins. Specifically, Tables 1 and 2 refer to old (from 1962 to 1977) and recent (from 1995 to 2005) articles, respectively, reporting adverse effects of polymyxins in patients without cystic fibrosis.

### Nephrotoxicity

#### Incidence

Although most of the studies or case reports published until 1983 did not include the definitions of nephrotoxicity, early reported experience with the use of polymyxins, mainly of colistin, revealed a high incidence of nephrotoxicity. The majority of the studies in the older literature referred to intramuscular administration of colistimethate sodium [4,19-25]. Notably, the incidence of nephrotoxicity was 36% in a study of patients with pre-existing acute or chronic renal disease and 20.2% in another large study of 288 patients [4,25]. Additionally, in three studies [26-28], intravenous colistimethate sodium was given for the treatment of patients with Gram-negative bacterial infections, including urinary tract infections, pneumonia, and septicaemia. These studies included 48, 23, and 8 patients, respectively; 10.5% of patients had prolonged increase of blood urea nitrogen levels (average increase of 50 mg/dl) [26], 26.1% of patients experienced renal impairment during therapy [27], and 50% had a fall in creatinine clearance (with a range of 16.5 to 38 ml/min) and an increase in serum creatinine levels (with a range of 0.2 to 2 mg/dl) [28]. Another interesting finding was the relatively high number of case reports that were published in the old literature reporting patients who experienced acute renal failure during treatment with colistimethate sodium. A point that deserves to be stressed, however, is that in most of these cases the total daily dose of colistimethate sodium was considerably higher compared to the currently recommended dose [3,29-34].

During the past seven years, colistimethate sodium has been re-introduced to clinical practice for the treatment of multidrug-resistant bacterial infections, mainly in the intensive care unit setting [9,10,12]. Data from recent studies do not corroborate the previously reported high incidence of polymyxin induced nephrotoxicity [11,35]. Although, the definition of nephrotoxicity was not standardized between the studies, two of them, which were conducted exclusively in intensive care units and used colistimethate sodium, reported that the observed nephrotoxicity was 14% [11] and 18.6% [12]. Notably, in one study that compared two therapeutic approaches – intravenous colistimethate sodium versus intravenous imipenem/cilastatin for the management of patients with ventilator-associated pneumonia due to *Acinetobacter baumannii*, nephrotoxicity occurred in 24% and 42% of patients, respectively [9]. Of note, polymyxin B was reported in the old literature to be associated with a relatively increased incidence of toxicity compared to colistimethate sodium. However, these data were not verified in two recent studies that showed that the incidence of nephrotoxicity was 14% [36] and 10% [37] among patients receiving polymyxin B therapy. Our experience is similar to that of the investigators of the previous studies [35,38].

#### Mechanisms

It has been suggested that the toxicity of polymyxins may be partly due to their D-amino acid content and fatty acid component. The proposed mechanism by which polymyxin B induces nephrotoxic events is by increasing membrane permeability, resulting in an increased influx of cations, anions, and water, leading to cell swelling and lysis [39,40]. An experimental study showed that colistin increased the transepithelial conductance of the urinary bladder epithelium [41]. The magnitude of the conductance's increase was dependent on concentration and length of exposure to polymyxins as well as the divalent cation concentration. The basic molecular mechanisms by which polymyxin B increases the transepithelial conductance in the urinary tract has been proposed to be the same as that of colistin [41]. Renal toxicity associated with the use of polymyxins is considered to be dose-dependent.

#### Clinical manifestations

Renal insufficiency, manifested by an increase in serum creatinine levels and decrease in creatinine clearance, represents a major adverse effect of the use of polymyxins. Occurrence of haematuria, proteinuria, cylindruria, or oliguria may also be associated with the administration of polymyxins. In addition, acute tubular necrosis can also develop [14]. Histological findings of colistin-induced renal damage usually involve focal irregular dilatation of tubules, epithelial and polymorphonuclear cell cast formation, and degeneration and regeneration of epithelial cells. In addition, separation of tubules by loose collagenous tissue, suggestive of edema, has also been reported. The basement membrane is usually intact, as well as the glomeruli [19,42].

#### Risk factors

Nephrotoxicity resulting from the use of colistimethate sodium appears to be less compared with that associated with polymyxin B. It is unclear whether there are independent factors that predispose patients to the development of nephrotoxic events. Children seem to experience less polymyxin-induced toxicity, probably in part because prescription of polymyxins, and generally all medications, is based on individual body weight in this patient population [4]. Concomitant administration of potential nephrotoxic agents, such as diuretics and some antimicrobial agents, increases the likelihood of development of renal adverse effects [4,43].

#### Treatment

When primary signs of renal dysfunction are present, early discontinuation of polymyxins is necessary. Quick diuresis by intravenously administered mannitol has also been proposed to enhance renal clearance of the drug and thus to reduce serum drug levels [32]. Meticulous supportive care, including close monitoring of fluid intake and output, frequent determinations of electrolytes, and appropriate management to maintain balance of fluids and electrolytes, is required when renal adverse effects of polymyxin use are detected. The influence of hemodialysis and peritoneal dialysis in decreasing serum levels of polymyxins has not been clarified. Old reports suggested that the amount of drug that is removed from blood by these two methods is relatively small [44,45]. Patients that underwent peritoneal dialysis lost approximately 1 mg of colistimethate sodium per hour [45]. Thus, in cases of polymyxin-induced renal failure, both therapeutic approaches have been used, not to decrease serum drug levels but in order to manage renal complications. Exchange transfusions have been proposed as an effective method for the removal of polymyxins [3].

### Neurotoxicity

#### Incidence

The incidence of neurotoxicity related to the use of polymyxins reported in the old literature was considerably less compared to nephrotoxicity. Specifically, the most frequently experienced neurological adverse effects were paresthesias that occurred in approximately 27% and 7.3% of patients receiving intravenous and intramuscular colistimethate sodium, respectively [4,26]. Furthermore, at least eight cases were published between 1964 and 1973 correlating the intramuscular administration of polymyxins with the development of episodes of respiratory apnea [22,33,46-51]. However, recently performed studies in patients without cystic fibrosis are not in accordance with the previously reported data regarding the incidence of polymyxin-induced neurotoxicity [11,12,38]. No episodes of neuromuscular blockade or apnea induced by polymyxins have been reported in the literature over the past 15 years or more.

#### Mechanisms

The interaction of polymyxins with neurons, which have a high lipid content, has been associated with the occurrence of several neurotoxic events. In addition, the probability of development of neurotoxicity has been directly associated with the concentration of the active form of polymyxins in the blood [14]. Neuromuscular blockade induced by polymyxins has been attributed to a presenaptic action of polymyxins that interferes with the receptor site and blocks the release of acetylcholine to the synaptic gap [33,52]. Other investigators have suggested a biphasic mechanism to explain this neurotoxic event; a short phase of competitive blockade between acetylcholine and polymyxins is followed by a prolonged phase of depolarization associated with calcium depletion [51,53,54]. Neurotoxicity resulting from the use of polymyxins is also considered to be dose-dependent.

#### Clinical manifestations

The reported neurological toxicity is associated with dizziness, generalized or not muscle weakness, facial and peripheral paresthesia, partial deafness, visual disturbances, vertigo, confusion, hallucinations, seizures, ataxia, and neuromuscular blockade. The last of these usually produces a myasthenia-like clinical syndrome, as well as respiratory failure or apnea due to respiratory muscle paralysis [33]. Paresthesias appear to be usually benign, and their mechanism seems to be unrelated to the interference with nerve transmission. An old study that assessed the safety of intramuscularly administered colistimethate sodium during 317 courses revealed that neurological adverse effects were manifested during the first four days of therapy in 83% of the patients who experienced neurotoxic events [4].

#### Risk factors

Risk factors that may potentially trigger the development of neurotoxicity include hypoxia and the co-administration of polymyxins with muscle-relaxants, narcotics, sedatives, anesthetic drugs, or corticosteroids [22,55]. A patient's gender may influence the likelihood of development of adverse effects. Specifically, neurotoxicity seems to be more common in women, although nephrotoxicity seems to be gender-independent [4]. Patients with impaired renal function or myasthenia gravis are at higher risk of developing neuromuscular blockade and respiratory paralysis [47].

#### Treatment

Mild neurological manifestations of polymyxins usually subside after prompt cessation of the drugs. In the presence of neuromuscular blockade, immediate discontinuation of polymyxins and other neurotoxic agents is also the first-line approach. Further management consists of mechanical respiratory support if apnea has been developed. The intravenous administration of calcium and cholinesterase inhibitors, such as neostigmine and edrophonium, has led to conflicting results [33,48]. Hemodialysis is indicated only in patients with co-existing acute renal failure.

### Other adverse events

#### Incidence

In studies published in the old literature, the reported incidence of allergic reactions related to colistimethate sodium use was approximately 2% [4]. Mild itching that did not require discontinuation of the drug was reported by approximately 22% of the patients receiving colistimethate sodium intravenously [27]. In addition, a few patients with episodes of rash were also reported [20,56]. In the recent literature, a few patients with episodes of contact dermatitis (eczema and erythematous eruption) have been reported in connection with topical use of colistin sulfate and ophthalmic administration of colistimethate sodium [57,58].

#### Mechanisms

Several milder adverse reactions, including pruritus, dermatitis, and drug fever, probably represent the result of the irritative effects of the active forms of polymyxins [14] and their histamine-releasing action, especially polymyxin B.

#### Clinical manifestations

Pruritus, contact dermatitis, macular rash or urticaria, ototoxicity, drug fever, and gastrointestinal disturbances may develop, although rarely, during treatment with polymyxins [26,57,59]. After intramuscular administration, pain may occur at the injection site [24]. Moreover, the development of pseudomembranous colitis represents a rare side effect of polymyxins. Intraventricular or intrathecal administration of polymyxins, especially in high doses, may lead to convulsions and signs of meningismus. During repeated ophthalmic application of polymyxin, low-grade conjunctivitis may develop [14].

An old case report suggested that the administration of colistimethate sodium intramuscularly in a patient with Gram-negative rod bacteremia was possibly associated with hepatotoxicity because an observed rise in serum glutamic oxaloacetic transaminase levels returned to normal after the drug was discontinued; in addition, post-mortem histological examination of the liver revealed non-specific changes (focal vacuolization of hepatic cells in the centrilobular fields with areas of focal necrosis), which were interpreted as drug-induced toxicity [19]. However, no other cases of liver toxicity have been reported in experimental or clinical studies on the use of polymyxins [38,60].

#### Risk factors

Patients with known allergy to bacitracin are also at higher risk of developing hypersensitivity reactions with the use of polymyxins, as cross-reaction between bacitracin and polymyxins exists [58].

#### Treatment

In most instances, withdrawal of polymyxins in combination with appropriate supportive treatment is adequate for the treatment of such adverse effects.

### Adverse events related to aerosolised colistin

Treatment with aerosolized colistin may be complicated by sore throat, cough, bronchoconstriction, and chest tightness. The nature of bronchoconstriction that develops during nebulization of polymyxins has been proposed to be associated with several mechanisms. Among them are direct chemical stimulation, the liberation of histamine, allergy in the airway, irritation from chemicals or from the foam that is produced during nebulization, and hyperosmolarity in the airway [61]. Nebulized polymyxins can cause bronchoconstriction even in patients with no history of asthma or atopy, although if these conditions exist the risk is greater [61]. Bronchoconstriction usually requires discontinuation of the medication, the administration of bronchodilators and supplemental oxygen.

### Prevention of adverse events

Early and correct adjustment of the dose of polymyxins in the presence of impaired renal function, frequent urinalyses and serum urea or creatinine measurements, close daily monitoring of urinary output and of neurological status, and the avoidance of concurrent administration of other agents with known nephrotoxicity or neurotoxicity may help prevent the development of adverse effects. Bronchoconstriction usually responds to treatment with bronchodilators; thus, pre-treatment of patients receiving inhaled colistimethate sodium with these medications could prevent the occurrence of this adverse event [61].

Recommendations regarding the dosage of polymyxins differ between various manufacturers. Colistin manufactured in the United States contains colistimethate sodium equivalent to 150 mg colistin base activity in each vial. The recommended dosage is 2.5 to 5 mg/kg per day, divided into 2 to 4 equal doses in adult patients with normal kidney function [62]. Manufacturers in the United Kingdom recommend a dosage of 4 to 6 mg/kg (50,000 to 75,000 IU/kg) intravenous colistimethate sodium per day, in 3 divided doses for adults and children with body-weight ≤ 60 kg, and 80 to 160 mg (1 to 2 million IU) every 8 hours for body-weight >60 kg [63].

The recommended dosage for intravenous polymyxin B sulphate is 1.5 mg to 2.5 mg/kg/day (15,000 IU to 25,000 IU/kg/day), divided into 2 equal doses for adults and children older than 2 years with normal renal function; 1 mg of polymyxin B is equal to 10,000 IU. Infants with normal renal function may receive up to 4 mg/kg/day (40,000 IU/kg/day) in cases of life-threatening infections [64].

### Overdoses

Overdoses with polymyxins, mainly with colistimethate sodium, have been reported several times in the old literature. Although, one case of a three year old child who received intramuscularly 450 mg (approximately 5.5 million IU) of colistimethate sodium reported no adverse effects, the majority of cases with polymyxin overdose resulted in acute renal failure and various manifestations of neurotoxicity, including neuromuscular blockade and apnea [3,31,33,34]. It should be emphasized that cases of polymyxin overdose with fatal consequences are scarce [29]. There is no antidote for polymyxin overdose. Management requires early cessation of the medication and appropriate supportive treatment. In the presence of established acute renal failure, haemodialysis and peritoneal dialysis can only manage renal complications, since they have little influence on the elimination of polymyxins, as discussed above. If apnea occurs, mechanical ventilation support is needed.

### Drug interactions

The concurrent use of polymyxins with curariform muscle relaxants and other neurotoxic drugs such as ether, tubocurarine, succinylcholine, gallamine, decamethonium, and sodium citrate must be avoided, since these agents may trigger the development of neuromuscular blockade [55]. Co-administration of sodium cephalothin and polymyxins may enhance the development of neurotoxicity, so this combination of antimicrobial medication should also be avoided [4]. In addition, antimicrobial agents with known neurotoxic effect, such as aminoglycosides, should generally be avoided or given with great caution in patients who receive polymyxins. In such instances, close monitoring of the patients receiving these antibiotics is mandatory. Experimental studies showed that application of polymyxins in combination with glutamic acid to a peripheral nerve could cause transgaglionic degenerative atrophy [65].

## Conclusion

The data from the recent literature suggest that the incidence of toxicity resulting from the use of polymyxins is less frequent and severe compared to what has been previously reported. Possible explanations for the observed discrepancy include the fact that the available formulation of colistimethate sodium for intramuscular administration was used intravenously in the old studies until a new formulation was prepared. In addition, the intramuscular formulation also contained dibucaine hydrochloride, which could potentiate the neurotoxic effect of colistimethate sodium. It should be highlighted that the dosages of polymyxins used in most of the studies published in the old literature were considerably higher compared to the recommended dosages administered nowadays. In fact, several reported cases of polymyxin-induced toxicity were associated with overdose. Thus, this may account for the observed difference in the incidence of polymyxin-induced toxicity noted between the old and recently published studies. A major limitation in the interpretation of polymyxin-induced nephrotoxicity and neurotoxicity in the intensive care unit setting, however, is the frequent existence of multiple organ failure, septic shock, and mechanical ventilation support. These conditions may considerably influence the assessment of polymyxin-induced toxicity. Dosage adjustment of polymyxins in the presence of impaired renal function and prompt discontinuation of polymyxins after development of early signs of their toxicity were not always performed in a timely fashion. Furthermore, the already reported experience regarding the toxicity of polymyxins in the old literature has led to more correct use of these antibiotics by physicians nowadays. In addition, the avoidance of co-administration of potential nephrotoxic and/or neurotoxic agents with polymyxins, as well as the development of critical care supplies, may also explain the observed differences. In the coming years further research is needed to assess the safety profile of polymyxins, clarify several aspects of their toxicity, and investigate the benefits of different dosing regimens, including the administration of these antibiotics in fewer daily doses.

## Key messages

• 	Polymyxins are valuable antibiotics for use in patients in the intensive care setting.

• 	Polymyxins have been recently re-introduced in clinical practice for the treatment of patients with multidrug-resistant Gram-negative bacterial infections.

• 	Nephrotoxicity and neurotoxicity represent the major adverse effects of polymyxins.

• 	Data from the recent literature suggest that the use of polymyxins is associated with lower and less severe toxicity compared to that reported in the old literature.

• 	Caution is needed when polymyxins are administered, particularly in patients with renal dysfunction.

## Competing interests

The authors declare that they have no competing interests.

## Authors' contributions

MEF conceived the study idea. Both authors contributed to the reviewing of the articles and writing of the manuscript. Both authors approved the final manuscript. MEF had full access to all the data in the study and takes responsibility for the integrity of the review of the data.

## Figures and Tables

**Table 1:** Old studies (from 1962 to 1977) reporting data on polymyxin-induced toxicity in patients without cystic fibrosis

	Year [ref]	Setting	Medication used	Number of patients	Demographics	Dosage of colistin/duration	Nephrotoxicity	Neurotoxicity	Other toxicities
1	1962 [26]	Medical wards	Colistimethate sodium (IV)	48		Adults: 150 mg q12 h Children: 5 to 10 mg/kg/day. Duration: at least 10 days	12 pts had transient mild elevation of BUN (average increase 14 mg/dl) and returned to normal. 5 pts had prolonged elevation of BUN (average increase 50 mg/dl) and returned to normal	13/48 pts paresthesias; 3/48 pts ataxia	3/48 pts pruritus. No drug fever, hepatic or bone marrow toxicity
2	1963 [19]	Medical wards	Colistimethate sodium (IM)	1	64 year old male	6.5 mg/kg/day (150 mg q8 h) for 12 days (he received concurrently kanamycin IM for 2 days and after colistin therapy chloramphenicol)	BUN increased from normal baseline values to 44 mg/dl (drug was stopped). The BUN continued to rise and then began to return to normal. Postmortem examination of the kidney revealed findings compatible with drug induced nephrotoxicity		Possible hepatotoxicity
3	1963 [66]	Medical and surgical wards	Colistimethate sodium (IM and topically)	62		Topically: 1% or 2% solution q4h or q12h. Duration (range): 2 to 7 d Intramuscularly (range): 150 to 300 mg/day. Duration (range): 1.5 to 19 d	Topically: no side effects	Topically: no side effects Intramuscularly: 15/55 pts reported one or more of the following: lethargy, dizziness, nausea, confusion, slurred speech, numbness, paresthesias, pruritus, pain at the injection	Topically: no side effects
4	1963 [20]	Medical wards	Colistimethate sodium (IM)	11		Dosage^a^: 1.5 MIU q12h for a week and continued for a further week if the pt was improving (2 pts received 2 MIU q8h for 5 days and then 3 MIU q8h)	No renal toxicity	2 pts trigeminal paresthesia	1 pt developed follicular rash of the face
				14		Dosage (range): 1 MIU q12h to 1.5 MIU q8 h for 7 or more days			
5	1964 [28]	Medical wards	Colistimethate sodium (IV)	8	Age (range): 25 to 69 years	Dosage: 2 to 2.5 mg/kg q12 h. Duration (range): 8 to 14 days.	4/8 pts fall in creatinine clearance (range: 16.5 to 38 ml/min) and increase in serum creatinine (range: 0.2 to 2 mg/dl)	No neurotoxicity	No pruritus
6	1964 [21]	Children's hospital	Colistimethate sodium (IM)	36 new-borns	Age (range): 6 hours to 12 days	Dosage (range): 2.5 to 5 mg/kg/day in 2 to 4 doses. Total dose (range): 10 to 240 mg (1 new-born (3.3 kg) received 160 mg of colistin (overdosage) in 7 days)	16 pts had renal epithelial tubular cells on urinalyses; 14 pts had urinary protein excretion	No neurotoxicity	
7	1964 [22]	Medical wards	Colistimethate sodium (IM)	1	50 year old male	Dosage: 300 mg/day for 5 days, then 200 mg/day for 4 days	Urinary retention, rise in blood urea nitrogen	Difficulty in breathing, dysphagia, generalized weakness, hallucinations, apnea requiring intubation	
8	1965 [50]	Medical wards	Colistimethate sodium (IM)	1	66 year old female with azotemia	Dosage: 150 mg q 12 h for 8 days. Cumulative dose: 2,550 mg		7th day of colistin: circumoral paresthesias; 8th day: vomiting, difficulty in breathing, moving, speaking, and became apneic; 10th day: grand mal seizures followed by transient right facial and arm weakness	
9	1965 [24]	Medical wards	Colistimethate sodium (IM)	17 (19 courses)	Age (range): 33 to 90 years	Total cumulative dose (range): 0.56 gr to 2.4 gr		8 pts dizziness – vertigo (1 pt discontinued), 5 pts oral paresthesias	3 pts pain at site of injection, 3 pts nausea/vomiting, 2 pts pruritus/rash
10	1965 [23]	Medical wards	Colistimethate sodium (IM)	1		75 mg q12 h		Episodes of ptosis, muscular weakness of the face and of the extremities	
11	1965 [25]	Medical wards (renal department)	Colistimethate sodium (IM)	25	12 males, 13 females. Age (range): 14 to 66 years. All with impaired renal function	Dosage (range): 2 MIU to 4.4 MIU/day. Duration (average): 8.5 days	9/25 pts had an increase in plasma creatinine levels		
12	1966 [46]	Medical wards	Colistimethate sodium (IM)	1	47 year old female	100 mg q8h		Perioral paresthesia, numbness in the hands, weakness, ataxia, lightheadedness, shortness of breath, apnea	Nausea, itching of the face, hands, and arms (no visible rash)
13	1966 [67]	Medical wards	Colistimethate sodium (IM)	21	All had urinary tract abnormalities or had undergone prostatectomy	Dosage: 120 mg (1.5 MIU) q8h for 7 days	No constant effect on creatinine clearance was observed		
14	1966 [43]	Medical wards	Colistimethate sodium (IM)	4 who developed acute renal failure	Age (range): 41 to 75 years. All with pre-existing renal disease	Dosage: 5 to 6.3 mg/kg/day. Duration (range): 3 to 12 days	Acute tubular failure (3 pts acute tubular necrosis, 1 pt recovered)	Retrosternal discomfort 1 pt	
15	1966 [48]	Medical wards	Colistimethate sodium (IM)	1	48 year old female	75 mg q12h (she also received chloramphenicol 500 mg q6h po)		Diplopia and bilateral eye ptosis, weakness of neck flexion, difficulty in raising her arms	
16	1966 [51]	Department of anaesthesiology	Colistimethate sodium (IM)	1	49 year old female with nephrolithiasis	75 mg q12 h (she also received chloramphenicol 500 mg q4h po and sulfisoxazole 1 g q4h po)		Post-operative apnea	
17	1967 [27]	Medical and surgical wards	Colistimethate sodium (IV)	23	Males, moderately to severely ill	Dosage (range): 1.1 to 5 mg/kg/day q12h for 6 to 7 days (in 2 cases the treatment was discontinued after 2 and 3 days)	6/23 pts renal impairment; 7/23 pts albuminuria	1 pt circumoral paresthesia	5/23 pts mild itching
18	1968 [44]	Medical wards	Colistimethate sodium (IV)	7	Age (range): 28 to 48 years. 4 females, 3 males; all had terminal and irreversible renal failure	2 to 3 mg/kg (1 dose)		2 pts mild dizziness and instability	
19	1968 [56]	Medical wards	Colistin sulfate (PO)	93 (48 cases *E. coli *and 45 cases *Shigella *spp.)		*E. coli *: 100,000 IU/kg/day in adults and 150,000 IU/kg/day in children for 7 days Shigella: 200,000 IU/kg/day in adults and 300,000 IU/kg/day in children for 8 to 10 days	No toxic symptoms	No toxic symptoms	1 pt generalized rash, 1 pt vomiting
20	1968 [49]	Medical wards (respiratory care unit)	Colistimethate sodium (IM) and Polymyxin B (IM or IV)	11	Age (range): 36 to 74 years. 4 females, 7 males; all had acute or chronic renal disease	Dosage of colistimethate sodium (range): 100 to 400 mg/day. Duration (range): 1 to 29 doses or 1 to 15 days. Dosage of polymyxin B: 50 mg (1 dose) IM (1 pt) and 100 mg (1 dose) IV (1 pt)		All pts at their admission had apnea that recovered in all cases. Paresthesias 2 pts, diplopia 3 pts, difficulty in swallowing 3 pts, ptosis 2 pts, generalized weakness 3 pts, blurring of vision 1 pt, slurred speech 1 pt, lethargy 1 pt, coma 1 pt	
21	1969 [42]	Medical wards	Colistimethate sodium (IV)	1	14 year old male with acute leukemia	Dosage: 5 mg/kg/day for 5 days, then increased to 7 mg/kg/day on day 6, 10 mg/kg/day on day 7, and 17 mg/kg/day on day 9. Duration: 14 days	Acute tubular necrosis		
22	1969 [29]	Medical wards (pediatrics)	Colistimethate sodium (IM)	1	4 year old female with appendicitis	Dosage: 30 mg/kg q6h (total dose received 1,050 mg during 42 h	Acute renal failure	Neuromyal hyperactivity, seizure-like episodes, uncoordination, disorientation, flaccid quadriplegia, respiratory arrest, apnea	
23	1970 [4]	Medical and surgical wards	Colistimethate sodium (IM)	288 (317 courses)		205 courses received a total of <1 gr, 69 courses 1 to 2 gr, 43 courses > 2 gr. All courses were administered IM q12 h	Total: 64/317 courses (renal insufficiency 63 pts, acute tubular necrosis 6 pts, hematuria 1 pt)	Total: 23/317 courses (paresthesias 15 pts, respiratory insufficiency and apnea 6 pts, nausea and vomiting 4 pts, dizziness 3 pts, muscular weakness 2 pts, peripheral neuropathy, confusion, psychosis, convulsive seizure 1 pt each)	Total allergic reactions: 7/317 (drug fever 3 pts, eosinophilia 2 pts, macular eruption 2 pts, urticarial eruption 1 pt)
24	1970 [68]	Medical wards and ICU	Colistimethate sodium (aerosol)	20	Age (range): 23 to 81 years	Group 1: 50 mg q8h for 7 days. Group 2: 100 mg q8h for 7 days	No toxic symptoms	1 pt experienced palpitations and a sensation of chest tightness (treatment was discontinued)	
25	1970 [3]	Department of pediatrics	Colistimethate sodium (IM)	1	Age: 10 months (male)	15 mg q6 h (2 doses) and then 250 mg (38.5 mg/kg) (3 dose)	Acute renal failure	No neurotoxicity	
26	1970 [30]	ICU, neurosurgical department	Colistimethate sodium (IV, IM, and aerosol)	14	Age (range): 31 to 71 years	Mean duration: 9.7 days. Dosage: 26 MIU/day: 10 MIU IM, 10 MIU IV, and 6 MIU aerosol	In all pts a considerable fall in creatinine clearance and rises in blood urea and serum creatinine levels were observed. 5 pts developed acute tubular necrosis (histological confirmed). In 6 pts renal function returned to normal		
27	1970 [69]	Department of renal disease	Colistimethate sodium (route of administration not reported)	1	41 year old	Duration: 7 days. Dose: 6.3 mg/kg/day	Severe oliguric renal failure		
28	1970 [31]	Department of pediatrics	Colistimethate sodium (IM)	1	3 year old	150 mg q8h (she received 3 injections)	No renal toxicity	No neurotoxicity	
29	1970 [32]	Medical wards (urology department)	Colistimethate sodium (IM)	1	33 year old male with a solitary kidney	25 mg q6h for 5 days and 250 mg q6h for 1 day	Increase in serum creatinine levels compared to baseline levels (1.1 mg/dl to 3 mg/dl) Returned to approximately normal values after 6 months	Muscular weakness, generalized paresthesias, speech disturbances, ptosis, hypotonia, areflexia, ataxia, difficulty in breathing	
30	1971 [47]	Department of neurology	Colistimethate sodium (IM)	1	70 year old male with myasthenia gravis	150 mg (one injection)		2 hours after the injection: muscular weakness; 30 minutes later he developed respiratory arrest	
31	1971 [70]	Department of respiratory diseases	Polymyxin B (aerosol)	2	Case 1: 51 year old female. Case 2: 57 year old male	Case 1: 15 mg Case 2: 10 mg		Case 1: pruritus, nausea, flashing, dyspnea Case 2: acute respiratory acidosis	
32	1973 [33]	Medical wards (Hemodialysis Centre)	Colistimethate sodium (IM)	2	Case 1: 16 year old female. Case 2: 23 year old female	Case 1: 150 mg q6h 1st day, 150 mg q4h 2nd day (20 mg/kg/day) Case 2: 180, 240, 180, 120 mg in divided doses on 1st, 2nd, 3rd, 4th day, respectively	Both pts developed acute renal failure	Case 1: neuromuscular blockade that resulted in quadriplegia, apnea, cardiac arrest Case 2: circumoral – acral paresthesias	
33	1974 [71]	Medical wards	Colistimethate sodium (IM)	1	66 year old male	6 MIU/day for 60 days	No renal toxicity	Total ophthalmoplegia, flaccid paralysis of both upper limbs, reduced speech fluency, difficulty in finding words, apathy	
34	1977 [34]	Department of pediatrics	Colistimethate sodium (IM)	1	5 year old male	200,000 IU/kg/day for 8 days	Acute oliguric renal failure	Muscular weakness, speech disturbances	

^a^1 mg of colistimethate sodium is approximately equal to 12,500 IU. BUN, blood urea nitrogen; ICU, intensive care unit; IM, intramuscularly; IV, intravenously; MIU, million international units; po, per os; Pt(s), patient(s); ref, reference.

**Table 2:** Recent studies (from 1995 to 2005) reporting data on polymyxin-induced toxicity in patients without cystic fibrosis

	Year [ref]	Setting	Medication used	Number. of patients	Demographics	Dosage of colistin/duration	Definition of nephrotoxicity	Nephrotoxicity	Neurotoxicity	Other toxicities
1	1995 [57]	Department of dermatology	Colistin sulfate (ointment/topically)	1	45 year old	50,000 IU for 10 days				Edematous eczema
2	1998 [58]	Department of dermatology	Colistimethate sodium (ophthalmic solution)	1	4 year old male with bilateral ocular prosthesis					After 3 weeks he developed itchy erythematous eruptions on both periorbital areas
3	1999 [72]	Neurosurgical wards	Colistimethate sodium (intraventricular)	2	16 year old male and 34 year old female	Case 1: 5 mg (62,500 IU) q12h for 19 days Case 2: 5 mg (62,500 IU) q12h for 5 days then 10 mg (125,000 IU) q12h for 12 days		No adverse reactions		
4	1999 [8]	ICU (52%), transplant unit (13%), surgical and medical wards (35%)	Colistimethate sodium (IV)	59 (60 cases)	Mean age: 42.1 years. Mean (± SD) APACHE II: 13.1 (± 7.0)	Mean duration: 12.6 d (2 to 34 d). Mean daily dose: 152.8 mg (approximately 2 MIU) (60–300 mg)		22 pts (37%; 11/41 with normal baseline renal function had worsening during treatment (mean increase in serum creatinine 0.9 ± 0.6 mg/dl) and 11/19 with abnormal baseline renal function had worsening during treatment (mean increase in serum creatinine 1.5 ± 1.4 mg/dl)). Nephrotoxicity did not cause discontinuation	No neuromuscular disorders	
5	2000 [73]	Medical wards	Colistimethate sodium (aerosol)	3	67 year old male, 45 year old male, 59 year old male	150 mg (2 MIU) q12h for 13 days, 100 mg (approximately 1.5 MIU) q12h for 14 days, 150 mg (2 MIU) q12h for 11 days		No nephrotoxicity		No hematological toxicity
6	2002 [74]	Neurosurgical wards	Colistimethate sodium (IV)	1	14 year old male	1 MIU q6h for 30 days		No adverse reactions		
7	2003 [10]	Abdominal organ transplantation ICU	Colistimethate sodium (IV)	23 (20 had received organ transplantation, 3 abdominal surgery)	Mean age: 52 years	Mean duration: 17 days (7 to 36 days)	Renal failure was defined by a requirement either for intermittent hemodialysis or for continuous venous hemofiltration	1/2 pts developed renal failure requiring artificial kidney support (the other 21 pts were already receiving artificial kidney support)	1 pt diffuse muscular weakness (resolved after discontinuation)	
8	2003 [11]	ICU	Colistimethate sodium (IV)	24 with sepsis, 26 courses of colistin	Mean age: 44.3 years. Mean APACHE II: 20.6	Mean duration: 13.5 days (4 to 24 days). Dosage: 3 MIU q8h	Renal failure was defined as an increase in serum creatinine >1 mg/dl during treatment	3 pts (14.3%). Only 1 pt required continuous venovenous hemodiafiltration	No clinically apparent neuromuscular transmission blockade	
9	2003 [36]	Tertiary care hospital	Polymyxin B (parenterally)	60 receiving polymyxin B	Mean age: 61 years	Mean duration: 13.5 days (1 to 56 days). Mean daily dose: 1.1 MIU	Renal failure was defined as doubling of serum creatinine value of ≥ 2.0 mg/dl	7/50 pts (14%)		
10	2003 [74]	ICU	Colistimethate sodium (IV)	35 (21 received colistin (CO group) and 14 imipenem (IM group))	Mean age: CO group 56.9 years, IM group 64.5 years. Mean APACHE II: CO group 19.6, IM group 20.5	CO group: mean duration 14,7 days (10 to 21 days). Dosage: 2.5 to 5 mg/kg/day	In patients with normal renal function (creatinine <1.2), renal failure was defined as creatinine value >2 mg/dl, as a reduction of creatinine clearance of 50% relative to antibiotic initiation, or need for renal replacement therapy. In patients with normal renal function, renal failure was defined as increase of 50% of the baseline creatinine level, as a reduction of creatinine clearance of 50% relative to antibiotic initiation, or need for renal replacement therapy	5/21 pts (24%; CO group), 6/14 pts (42%; IM group)		
11	2004 [75]	ICU	Colistimethate sodium (IV)	1	41 year old male	2 MIU/day continuous infusion		No adverse reactions		
12	2004 [76]	ICU	Colistimethate sodium (IV)	1	48 year old male	9 MIU/day (2.5 mg/kg/day) for 15 days		No adverse reactions		
13	2004 [37]	Tertiary care hospital, ICU (92%)	Polymyxin B (IV)	25 (29 courses: 21 IV, 6 aerosol, 2 combination)	Mean age: 55 years. Mean APACHE II: 21	Loading dose on day 1 with 2.5 to 3 mg/kg IV polymyxin B. Aerosolized: approximately 2.5 mg/kg/day (approximately 1.75 MIU). Mean duration: 19 d (2 to 57 d)	Nephrotoxicity was defined as the doubling of serum creatinine during therapy	3/29 courses (10%)	2/29 courses (7%) onset of seizures and neuromuscular weakness possibly related to polymyxin B	
14	2005 [12]	ICU	Colistimethate sodium (IV)	43	Mean age: 56.5 years. APACHE II: 25.8 ± 3.7	3 MIU q8h	Acute renal failure was defined as a rise of ≥ 2 mg/dl in serum creatinine level in patients with previously normal renal function. In patients with a history of renal insufficiency, acute on chronic renal failure was defined as at least doubling of the baseline serum creatinine level (defined as the creatinine level at the initiation of colistin treatment)	8/43 pts (18.6%; 3/35 pts with normal renal function (8.6%) and 5/8 pts with chronic renal failure (62.5%))	No paresthesias, vertigo, muscle weakness, or apnea were observed	
15	2005 [77]	ICU (84%), medical (11%), surgical (5%)	Colistimethate sodium (aerosol, IV, IM, intrathecal)	80 (85 courses: 71 aerosol, 12 IV or IM, 2 intrathecal)	Mean age: 57 ± 15 years	Mean duration of aerosol: 12 ± 8 d. Mean duration of IV or IM: 11 ± 6 d. Mean duration of intrathecal: 8 d and 10 d	Nephrotoxicity was defined as a serum creatinine increase of 50% or 1 mg/dl with respect to the baseline level during treatment	12 courses of IV or IM were recorded. Mean ± SD baseline serum creatinine: 1.25 ± 0.79 mg/dl. Mean ± SD final serum creatinine: 1.20 ± 0.64 mg/dl. Mean ± SD baseline BUN: 8.95 ± 8.96 μmol/l. Mean ± SD final BUN: 8.39 ± 8.06 μmol/l		
16	2005 [38]	Mainly ICU pts	Colistimethate sodium (IV)	17 (19 courses)	Median age: 51 years. Median APACHE II: 14	Mean ± SD duration: 43.4 ± 14.6 days. Mean ± SD daily dose: 4.4 MIU (352 mg) ± 2.1 MIU (168 mg)	Renal failure was defined as an increase more than 50% of the baseline creatinine level to a value higher than 1.3 mg/dl or as a decline in renal function requiring renal replacement therapy	Median baseline serum creatinine: 0.6 mg/dl. Slight increase of the median of values of creatinine at the end by 0.1 mg/dl. Median baseline BUN: 42 mg/dl. Median final BUN: 41 mg/dl. 1 pt had an increase of more than 50% of the baseline creatinine level to a value higher than 1.3 mg/dl at the end of colistin treatment	No apnea or other evidence of neuromuscular blockade. 1 pt polyneuropathy (improved after the end of colistin treatment)	No hepatobiliary toxicity
17	2005 [35]	ICU (80%), medical and surgical wards (20%)	Colistimethate sodium (IV)	50 (54 episodes)	Mean age: 59.2 years. Mean APACHE II: 16.1	Mean duration: 21.5 days. Mean daily dose: 4.5 MIU	Renal failure was defined as an increase more than 50% of the baseline creatinine level to a value higher than 1.3 mg/dl or as a decline in renal function requiring renal replacement therapy	4/50 pts (8%)	1 pt polyneuropathy (not confirmed) resolved without discontinuation	
18	2005 [78]	ICU	Colistimethate sodium (aerosol)	8	Mean age: 59.6 years. Mean APACHE II: 14.6	Dosage (range): 1.5 to 6 MIU/day. Duration (mean): 10.5 days		Worsening of renal function: 1 pt	No neurotoxicity	
19	2005 [79]	ICU	Colistimethate sodium (IV)	1	57 year old male	250 mg q6h for 4 days		Acute renal failure (on the 4th day of colistin therapy)		
20	2005 [80]	Neurosurgical wards	Colistimethate sodium (intraventricular)	1	23 year old female	125,000 IU q12h for 3 weeks		No adverse reactions		
21	2005 [81]	ICU	Colistimethate sodium (IV)	55	Mean age: 40 ± 16 years. Mean APACHE II: 21 ± 7	Duration (mean): 13 ± 5 days	Renal failure was defined as a serum creatinine value of 2 mg/dl or higher, as a reduction in creatinine clearance of 50% compared to therapy initiation, or as a decline in renal function that prompted renal replacement therapy	No adverse reactions. Mean creatinine levels before treatment: 2.3 ± 0.5 mg/dl. Mean creatinine levels after treatment: 2.5 ± 0.6 mg/dl		
22	2005 [82]	ICU	Colistin (IV)	1	35 year old male	6 MIU/day for 12 days, 3 days, and 1 day		Acute renal failure occurred at the 2nd and 3rd introduction of colistin. Renal function returned to normal values within 3 and 5 days after colistin withdrawal		
23	2005 [83]	ICU	Colistimethate sodium (IV)	14	Mean age: 49 years	Mean dose: 6 MIU/day. Mean duration: 12 days		1 pt experienced deterioration of renal function (serum creatinine up to 2.8 mg/dl)		
24	2005 [84]	ICU, medical wards	Colistimethate sodium (aerosol)	21	Mean age: 60.6 ± 15 years. Mean APACHE II: 23.1 ± 9.1	19 pts received 2 MIU/day, 1 pt 3 MIU/day, and another pt 4 MIU/day. Median duration: 14 days	Renal failure was defined as a decrease in the estimated creatinine clearance rate of 50%, compared with the rate at the start of therapy, or a decline in renal function that necessitated renal replacement therapy	No episodes of acute renal failure	No symptoms of neurotoxicity	1 pt experienced bronchospasm that resolved on discontinuation of colistin therapy

BUN, blood urea nitrogen; ICU, intensive care unit; IM, intramuscularly; IV, intravenously; MIU, million international units; Pt(s), patient(s); ref, reference; SD, standard deviation.
